# Role of Surrogate Markers of Atherosclerosis in Clinical and Subclinical Thyroidism

**DOI:** 10.1155/2012/109797

**Published:** 2012-02-23

**Authors:** Mehmet Gunduz, Ercan Gunduz, Fatih Kircelli, Nazan Okur, Mesut Ozkaya

**Affiliations:** ^1^Department of Internal Medicine, Kahramanmaras University, 46000 Kahramanmaras, Turkey; ^2^Department of Internal Medicine, Malatya Hekimhan State Hospital, 44400 Malatya, Turkey; ^3^Division of Nephrology, Yozgat State Hospital, 66000 Yozgat, Turkey; ^4^Department of Radiology, Kahramanmaras University, 46000 Kahramanmaras, Turkey

## Abstract

*Background*. Data on the relationship between homocysteine, plasminogen activator inhibitor 1, hs-CRP, fibrinogen, and carotid intima media thickness (CA-IMT) is plenty but contradicting and the majority of the studies investigated this issue in only specific thyroidism groups. The aim of this paper was to investigate these relations in patients with subclinical and clinical hypo- and hyperthyroidism. *Methods*. In this cross-sectional study, 16 patients from each thyroidism group and 20 healthy cases were enrolled. Fibrinogen levels and plasminogen activator inhibitor 1 (PAI-1) activity were assessed. CA-IMT was determined by gray-scale high-resolution color Doppler ultrasound. *Results*. Serum homocysteine levels were higher in hypothyroidic patients compared to the control (*P* = 0.003). Fibrinogen levels were higher in patients with subclinical hypothyroidism compared to other groups (*P* < 0.05). There was no difference between groups regarding PAI-1. Whereas total cholesterol, homocysteine, and LDL were correlated with CAIMT, hs-CRP, PAI-1, and fibrinogen were not. In the clinical hypothyroidism group, the correlation of homocysteine with CA-IMT was derived from the correlation between CA-IMT and homocysteine. *Conclusions*. Homocysteine and fibrinogen levels are higher in patients with clinical and subclinical hypothyroidism, respectively. Homocysteine level is associated with CA-IMTonly in patients with clinical hypothyroidism.

## 1. Introduction

Thyroid diseases are among the most common endocrinological disorders. Depending on the levels of thyroid hormones, it may present either as normo-, hypo-, or hyperthyroidism. It can also be classified as subclinical (normal Thyroid stimulating hormone-TSH) or clinical (abnormal TSH). Subclinical hypothyroidism is the most common form.

Disturbances in thyroid metabolism affect various organs such as the heart, vessels, and kidney [1–6]. With altered thyroid functions, changes in oxygen demand, the contractility of the heart, cardiac output, and systemic vascular resistance lead to a hectic environment and worsening cardiovascular outcomes [7–9].

Cardiovascular diseases are the main causes of death in the world. Atherosclerosis is a major contributor to this problem. Carotid intima media thickness (CA-IMT) measurement by Doppler USG is a validated method to determine extent of atherosclerosis [10, 11]. Age, male sex, smoking, diabetes, hypertension, and dyslipidemia are among the well-known risk factors for atherosclerosis. Also, several other risk factors such as obesity, sedentary lifestyle, high hs-CRP, homocysteine, lipoprotein, and plasminogen activator inhibitor 1 (PAI-1) have been linked to atherosclerotic cardiovascular diseases [12]. Accelerated atherosclerosis is well known in autoimmune rheumatic diseases compared to other autoimmune diseases such as thyroid autoimmunity [13, 14].

Thyroid hormones via blood vessel dilatation, production of vasodilator molecules, inhibition of angiotensinogen II receptor expression, and its signal transduction regulate endothelial function and vascular homeostasis and have anti-atherosclerosis effects [15–18]. Regarding thyroid hormones, both clinical and subclinical hypothyroidism has been linked to atherosclerosis [19, 20]. Moreover, it has been shown that thyroid hormone replacement therapy leads to regression of subclinical hyperthyroidism [21–24]. A major contributor to this problem is the hyperlipidemia observed in these patients. Data on the association between hyperthyroidism and atherosclerosis is less clear.

With this background in mind, our aim was to investigate the extent of atherosclerosis in subclinical and clinical hypo- and hyperthyroidism along with potential roles of homocysteine and PAI-1 in an otherwise healthy population.

## 2. Materials and Methods

### 2.1. Patients

Of the first two patients who applied to the endocrinology outpatient clinic with symptoms and/or signs of thyroid dysfunction each day and met the inclusion and exclusion criteria below were stratified into the corresponding thyroidism groups according to their thyroid function results (subclinical and clinical hypothyroidism, subclinical and clinical hyperthyroidism). Each group was planned to consist of 16 patients. Group 1 included patients with high TSH and normal fT3 and fT4 levels (subclinical hypothyroidism), group 2 high TSH and low fT3 and fT4 levels (clinical hyperthyroidism), group 3 low TSH and normal fT3 and fT4 levels (subclinical hyperthyroidism), and group 4 low TSH and now fT3 and fT4 levels (clinical hyperthyroidism). Also, 20 random people without any thyroid hormone abnormality were used as control.

Inclusion criteria were to be aged between 18 and 65 years, to have thyroid hormone dysfunction assessed by laboratory findings, to have a body mass index of 20–30 kg/m^2^, to have no history of any kind of thyroid dysfunction, and not to be on thyroid hormone replacement. Exclusion criteria were to have history of diabetes, hypertension, coronary artery disease, and familial hypercholesterolemia, to be on lipid lowering drugs, and to have body mass index <20 kg/m^2^ or >30 kg/m^2^.

Demographical, clinical, and laboratory findings were recorded from patients' charts. Routine biochemical parameters were measured at our university's core biochemistry laboratory. fT3, fT4, and TSH were measured by chemiluminescence method by an automated analyzer (Immulite 2000, Siemens, Germany). Normal ranges for fT3 were 1.8–4.2 pg/mL, for fT4 0.8–1.9 ng/dL, and for TSH 0.4–4 mU/mL. Fibrinogen levels were measured nephelometrically according to the manufacturer's protocol by using a commercially available kit on Behring BCS coagulation analyzer (Dade Bering, Schwalbach, Germany). Plasminogen activator inhibitor 1 (PAI-1) activity was measured according to the manufacturers' protocol with indirect chromogenic assay by using SPECTROLYSE PAI-1 ELISA kit (American Diagnostics, USA).

The study protocol was approved by local ethics committees and informed consent was obtained from each patient. The study was performed according to the recommendations of the Declaration of Helsinki.

### 2.2. Measurement of CA-IMT

Ultrasonographic studies on common carotid arteries were carried out by gray-scale high-resolution color Doppler ultrasound (Prosound SSD- 3500 SV ALOKA, Zug, Switzerland) equipped with 12 MHz linear transducer. The same operator performed all procedures on both sides of two longitudinal images of the each common carotid artery on the morning in supine position. Average of the two CA-IMT (proximal and distal) values from each side was used to calculate mean CA-IMT on each side. Intraobserver coefficient of variation was 2.21%.

### 2.3. Statistical Analysis

All results are reported as mean ± SD. *P* value less than 0.05 was considered as statistically significant. Comparisons between two groups were assessed with the Student's unpaired *t*-test or Mann-Whitney test, as appropriate. Pearson's and Spearman's rank correlations were used to assess correlations of fibrinogen, homocysteine, hs-CRP, and PAI-1 with left and right CA-IMT. Correlation was analyzed both in the whole population except control and also in the subgroups. All statistical analyses were performed using SPSS, version 15 (Chicago, IL, USA).

## 3. Results

### 3.1. Subclinical Hypothyroidism

Mean age was 40.8 ± 11.8 years and 81.3% were female. Mean fT3, fT4, and TSH levels were 3.02 ± 0.67 pg/mL, 1.13 ± 0.24 ng/mL, and 6.91 ± 1.74 IU/mL. Mean homocysteine level was 12.76 ± 3.11 mmol/L, hs-CRP 5.98 ± 7.84 mg/L, and PAI-1 52.21 ± 31.93 ng/mL. Mean CA-IMT was 0.61 ± 0.11 mm.

### 3.2. Clinical Hypothyroidism

Mean age was 38.0 ± 10.2 years and 87.5% were female. Mean fT3, fT4, and TSH levels were 2.52 ± 0.92 pg/mL, 0.76 ± 0.29 ng/mL, and 29.87 ± 23.65 IU/mL. Mean homocysteine level was 15.05 ± 9.87 mmol/L, hs-CRP 5.41 ± 3.48 mg/L, fibrinogen 283.00 ± 81.16 mg/dL, and PAI-1 42.78 ± 33.65 ng/mL. Mean CA-IMT was 0.62 ± 0.13 mm.

### 3.3. Subclinical Hyperthyroidism

Mean age was 41.6 ± 16.43 years and 81.3% were female. Mean fT3, fT4, and TSH levels were 3.46 ± 0.53 pg/mL, 1.33 ± 0.18 ng/mL, and 0.12 ± 0.09 IU/mL. Mean homocysteine level was 11.93 ± 3.72 mmol/L, hs-CRP 3.80 ± 1.72 mg/L, fibrinogen 275.37 ± 68.42 mg/dL, and PAI-1 53.83 ± 37.21 ng/mL. Mean CA-IMT was 0.58 ± 0.17 mm.

### 3.4. Clinical Hyperthyroidism

Mean age was 41.6 ± 11.8 years and 68.8% were female. Mean fT3, fT4, and TSH levels were 6.7 ± 3.33 pg/mL, 2.90 ± 1.70 ng/mL, and 0.01 ± 0.02 IU/mL. Mean homocysteine level was 10.23 ± 3.13 mmol/L, hs-CRP 3.93 ± 1.91 mg/L, fibrinogen 275.37 ± 68.42 mg/dL, and PAI-1 53.83 ± 37.21 ng/mL. Mean CA-IMT was 0.59 ± 0.15 mm.

### 3.5. Comparison of Groups and Correlations

As would be expected, patients with subclinical hypothyroidism had higher BMI compared to the control (*P* = 0.035). This was more pronounced in patients with clinical hypothyroidism (*P* = 0.008). A similar trend was true for total cholesterol and LDL levels. Comparison of the clinical and demographical parameters of the groups is shown in Table 1.

Serum homocysteine levels were significantly higher in hypothyroidic patients compared to the control (*P* = 0.003). There was no difference between hypo- and hyperthyroidic patients. Fibrinogen levels were higher in patients with subclinical hypothyroidism compared to other groups (*P* < 0.05). There was no difference between groups regarding PAI-1. Comparison of the clinical and demographical parameters of the groups is presented in Table 2.

**Figure 1:**
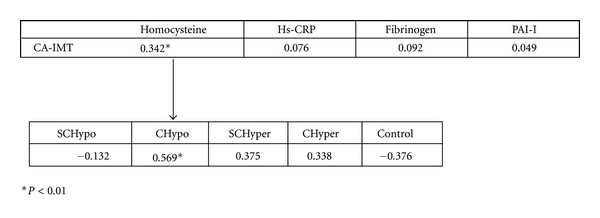
Correlations between homocysteine, hs-CRP, fibrinogen, PAI-1, and CA-IMT and correlations of homocysteine and CA-IMT between thyroidism groups. PAI-I: plasminogen activator inhibitor-1; CA-IMT: carotid intima media thickness; SCHypo: subclinical hypothyroidism; CHypo: clinical hypothyrodism; SCHypo: subcilinical hyperthyroidism; CHyper: clinical hyperthyroidism.

Whereas total cholesterol, LDL, and homocysteine were correlated with CA-IMT, hs-CRP, PAI-1, and fibrinogen were not (Figure 1). The correlation of homocysteine with CA-IMT was derived from the strong correlation in the clinical hypothyroidism group (Figure 1).

## 4. Discussion

In this study, we provide, for the first time, data on the association between several novel surrogate cardiovascular markers (hs-CRP, fibrinogen, homocysteine, and PAI-1) and CA-IMT in patients with clinical/subclinical hypo- and hyperthyroidism. Only homocysteine level was associated with CA-IMT and this was derived from the strong association between homocysteine and CA-IMT in patients with clinical hypothyroidism.

Data on PAI-1 in thyroid diseases is controversial. Erem et al. reported higher levels patients with hypothyroidism compared to the controls [25, 26]. This was indeed linked to higher risk of peripheral thrombosis [25]. Contrary, another study found lower levels in patients with subclinical hypothyroidism [27]. In other studies, there was no difference in PAI-1 levels in patients with subclinical hypothyroidism compared to the control [28, 29]. In hyperthyroidic patients, higher levels in patients with hyperthyroidism were reported [30, 31]. Our results confirm no difference between hypo- and hyperthyroidism groups and also with control. Also, we report that in these patients PAI-1 levels are not related to CA-IMT, a marker of atherosclerosis. We also did not find any association between BMI and PAI-1 levels, which is proposed as a production site for PAI-1 in any of the groups [32].

Hyperhomocysteinemia is proposed as a contributor to atherosclerosis by stimulating oxidation of LDL and causing endothelial dysfunction [33–35]. Both hypothyroidism and hyperthyroidism are associated with alterations in homocysteine levels [36–38]. It is suggested that levels are increased in hypothyroidic patients and are decreased in hyperthyroidic patients [39]. Hypothyroidism has been proposed to decrease hepatic levels of enzymes involved in homocysteine metabolism. Also alteration of LDL in hypothyroidism makes it plausible. In the New Mexico Elder Health survey, no difference in patients with subclinical hypothyroidism (*n* = 112) compared to patients with TSH ≤ 4.6 mcu/mL (643) was observed [40]. Our study confirms the finding that levels are higher in clinically hypothyroidic patients compared to control and not different from the control in patients with subclinical hypothyroidism. In line with the previous literature, levels are lower in hyperthyroidic patients compared to hypothyroidic patients but are not statistically different than the control.

The association between homocysteine levels and CA-IMT is not clear [41–43]. Data on this topic is almost nonexistent in patients with thyroid disease. Our results show a positive correlation between homocysteine levels and CA-IMT derived from the strong correlation between these 2 parameters in patients with clinical hypothyroidism.

Several studies have reported a positive association between CRP levels and cardiovascular events and atherosclerosis [44–47]. However causality of CRP on atherosclerosis remains unclear. It is suggested that CRP may be a reflection of the inflammation caused by smoking, obesity, metabolic syndrome, and other classical cardiovascular risk factors [48, 49]. Various studies have reported contradicting results on association between CRP levels and severity and progression of atherosclerosis [50–53].

In patients with hypothyroidism data on the level of CRP is contradictory [54–58]. In a study by Christ-Crain et al., despite no association between thyroid hormone levels and CRP, levels were found higher in hypothyroidic patients, but thyroid hormone replacement did not have any effect on CRP levels in patients with subclinical hypothyroidism [59]. A similar finding was observed in the study by Lee et al. In our study, levels were higher in patients with subclinical and clinical hypothyroidism compared to other groups, but yet not statistically significant. Also, no relation with CA-IMT was present. Possible explanation for these results may be the differences in the study population, as well as the sample size. Our results also confirm the previous data that CRP levels are not different from controls in hyperthyroidic patients [60, 61].

The strongest side of our study is comparison between thyroidism subgroups along with a comparison with a control. The major limitation of our study is its cross-sectional nature and measurement at one time-point. Despite most of the studies in the literature have enrolled less than 30 patients in comparison studies, the number of patients in each group in our study may still be considered relatively small. A further study enrolling higher number of patients and analyzing the value of these parameters in a time-dependent manner may yield more data on this issue.

As a conclusion, our results show that homocysteine and fibrinogen levels are higher in patients with clinical and subclinical hypothyroidism, respectively. Homocysteine level was associated with CA-IMT in patients with thyroidism, and this was derived from the strong association between homocysteine and CA-IMT in patients with clinical hypothyroidism. Determination of homocysteine in clinical hypothyroidic patients may provide information on the cardiovascular burden. Also, therapy to reduce homocysteine levels may be an important approach in these patients to decrease the cardiovascular burden.

## Figures and Tables

**Table 1:** Clinical and demographical characteristics of the patient population.

	Subclinical hypothyroidism	Clinical hypothyroidism	Subclinical hyperthyroidism	Clinical hyperthyroidism	Control
Gender (F/M)	13/3	14/2	13/3	11/5	9/11
Age (years)	40,8 ± 11,8	38,0 ± 10,2	41,6 ± 16,4	41,6 ± 11,8	32,8 ± 5,7
BMI (kg/m^2^)	26,72 ± 2,32*	27,14 ± 2,25*	24,65 ± 3,07	24,48 ± 1,84	24,34 ± 2,43
MAP (mmHg)	88,8 ± 12,0	86,4 ± 9,6	84,9 ± 9,7	88,2 ± 10,6	80,0 ± 11,7
TSH (ìIU/mL)	6,91 ± 1,74*	29,87 ± 23,65*	0,12 ± 0,09*	0,01 ± 0,02*	1,39 ± 0,68*
sT3 (pg/mL)	3,02 ± 0,67	2,52 ± 0,92	3,46 ± 0,53	6,7 ± 3,33	3,22 ± 0,97
sT4 (ng/dL)	1,13 ± 0,24	0,76 ± 0,29	1,33 ± 0,18	2,90 ± 1,70	1,4 ± 0,19
T.cholestrol (mg/dL)	204 ± 41*	207 ± 69*	181 ± 28	147 ± 40	164 ± 27
Triglyceride (mg/dL)	186 ± 127	141 ± 28	158 ± 76	105 ± 57	104 ± 44
HDL (mg/dL)	41 ± 8	43 ± 7	41 ± 6	41 ± 10	42 ± 11
LDL (mg/dL)	126 ± 34*	135 ± 63*	110 ± 28	85 ± 31	111 ± 41
CA-IMT (mm)	0,61 ± 0,11	0,62 ± 0,13	0,58 ± 0,17	0,59 ± 0,15	0,53 ± 0,08

*P < 0.05.

F: female; M: male; BMI: body mass index; MAP: mean arterial pressure; TSH: thyroid stimulating hormone; HDL: high-density lipoprotein; LDL: low-density lipoprotein; CA-IMT: carotid intima media thickness.

**Table 2:** Homocysteine, hs-CRP, Fibrinogen, and PAI-1 levels in Thyroidism groups.

	SCHypo	CHypo	SCHyper	CHyper	Control
Homocysteine (mmol/L)	12,76 ± 3,11	15,05 ± 9,87	11,93 ± 3,72	10,23 ± 3,13	8,41 ± 3,86
Hs-CRP (mg/L)	5,98 ± 7,84	5,41 ± 3,48*	3,80 ± 1,72	3,93 ± 1,91	3,48 ± 0,64
Fibrinogen (mg/dL)	327,56 ± 100,84*	283,00 ± 81,16	275,37 ± 68,42	273,56 ± 76,08	271,50 ± 52,47
PAI-1 (ng/mL)	52,21 ± 31,93	42,78 ± 33,65	53,83 ± 37,21	64,19 ± 34,38	54,06 ± 33,78

*P < 0.05.

PAI-1: plasminogen activator inhibitor-1; SCHypo: subclinical hypothyroidism; CHypo: clinical hypothyroidism; SCHyper: subclinical hyperthyroidism; CHyper: clinical hyperthyroidism.

## References

[1] Rodondi N, Newman AB, Vittinghoff E (2005). Subclinical hypothyroidism and the risk of heart failure, other cardiovascular events, and death. *Archives of Internal Medicine*.

[2] Vanhaelst L, Neve P, Chailly P, Bastenie PA (1967). Coronary-artery disease in hypothyroidism. Observations in clinical myxoedema. *The Lancet*.

[3] Cini G, Carpi A, Mechanick J (2009). Thyroid hormones and the cardiovascular system: pathophysiology and interventions. *Biomedicine and Pharmacotherapy*.

[4] Ichiki T (2010). Thyroid hormone and atherosclerosis. *Vascular Pharmacology*.

[5] Lim VS (2001). Thyroid function in patients with chronic renal failure. *American Journal of Kidney Diseases*.

[6] van Hoek I, Daminet S (2009). Interactions between thyroid and kidney function in pathological conditions of these organ systems: a review. *General and Comparative Endocrinology*.

[7] Klein I, Ojamaa K (2001). Thyroid hormone and the cardiovascular system. *The New England Journal of Medicine*.

[8] Park KW, Dai HB, Ojamaa K, Lowenstein E, Klein I, Sellke FW (1997). The direct vasomotor effect of thyroid hormones on rat muscle resistance arteries. *Anesthesia and Analgesia*.

[9] Ojamaa K, Klemperer JD, Klein I (1996). Acute effects of thyroid hormone on vascular smooth muscle. *Thyroid*.

[10] O’Leary DH, Polak JF (2002). Intima-media thickness: a tool for atherosclerosis imaging and event prediction. *American Journal of Cardiology*.

[11] Baldassarre D, Tremoli E, Amato M, Veglia F, Bondioli A, Sirtori CR (2000). Reproducibility validation study comparing analog and digital imaging technologies for the measurement of intima-media thickness. *Stroke*.

[12] Ridker PM, Stampfer MJ, Rifai N (2001). Novel risk factors for systemic atherosclerosis: a comparison of C-reactive protein, fibrinogen, homocysteine, lipoprotein(a), and standard cholesterol screening as predictors of peripheral arterial disease. *JAMA*.

[13] Gremese E, Ferraccioli G (2011). The metabolic syndrome: the crossroads between rheumatoid arthritis and cardiovascular risk. *Autoimmunity Reviews*.

[14] Sarzi-Puttini P, Atzeni F, Gerli R (2010). Cardiac involvement in systemic rheumatic diseases: an update. *Autoimmunity Reviews*.

[15] Klein I, Ojamaa K (2001). Thyroid hormone: targeting the vascular smooth muscle cell. *Circulation Research*.

[16] Napoli R, Biondi B, Guardasole V (2001). Impact of hyperthyroidism and its correction on vascular reactivity in humans. *Circulation*.

[17] Taddei S, Caraccio N, Virdis A (2003). Impaired endothelium-dependent vasodilatation in subclinical hypothyroidism: beneficial effect of levothyroxine therapy. *The Journal of Clinical Endocrinology and Metabolism*.

[18] Fukuyama K, Ichiki T, Takeda K (2003). Downregulation of vascular angiotensin II type 1 receptor by thyroid hormone. *Hypertension*.

[19] Cappola AR, Ladenson PW (2003). Hypothyroidism and atherosclerosis. *The Journal of Clinical Endocrinology and Metabolism*.

[20] Hak AE, Pols HAP, Visser TJ, Drexhage HA, Hofman A, Witteman JCM (2000). Subclinical hypothyroidism is an independent risk factor for atherosclerosis and myocardial infarction in elderly women: the Rotterdam study. *Annals of Internal Medicine*.

[21] Kim SK, Kim SH, Park KS, Park SW, Cho YW (2009). Regression of the increased common carotid artery-intima media thickness in subclinical hypothyroidism after thyroid hormone replacement. *Endocrine Journal*.

[22] Kebapcilar L, Comlekci A, Tuncel P (2010). Effect of levothyroxine replacement therapy on paraoxonase-1 and carotid intima-media thickness in subclinical hypothyroidism. *Medical Science Monitor*.

[23] Adrees M, Gibney J, El-Saeity N, Boran G (2009). Effects of 18 months of l-T4 replacement in women with subclinical hypothyroidism. *Clinical Endocrinology*.

[24] Fadeyev VV, Sytch J, Kalashnikov V, Rojtman A, Syrkin A, Melnichenko G (2006). Levothyroxine replacement therapy in patients with subclinical hypothyroidism and coronary artery disease. *Endocrine Practice*.

[25] Erem C, Kavgaci H, Ersöz H (2003). Blood coagulation and fibrinolytic activity in hypothyroidism. *International Journal of Clinical Practice*.

[26] Chadarevian R, Bruckert E, Leenhardt L, Giral P, Ankri A, Turpin G (2001). Components of the fibrinolytic system are differently altered in moderate and severe hypothyroidism. *The Journal of Clinical Endocrinology and Metabolism*.

[27] Rennie JAN, Bewsher PD, Murchison LE, Ogston D (1978). Coagulation and fibrinolysis in thyroid disease. *Acta Haematologica*.

[28] Jorde R, Figenschau Y, Hansen J-B (2006). Haemostatic function in subjects with mild subclinical hypothyroidism. The Tromsø study. *Thrombosis and Haemostasis*.

[29] Müller B, Tsakiris DA, Roth CB, Guglielmetti M, Staub JJ, Marbet GA (2001). Haemostatic profile in hypothyroidism as potential risk factor for vascular or thrombotic disease. *European Journal of Clinical Investigation*.

[30] Wahrenberg H, Wennlund A, Hoffstedt J (2002). Increased adipose tissue secretion of interleukin-6, but not of leptin, plasminogen activator inhibitor-1 or tumour necrosis factor alpha, in Graves’ hyperthyroidism. *European Journal of Endocrinology*.

[31] Akinci B, Comlekci A, Yener S (2007). Thrombin activatable fibrinolysis inhibitor antigen levels are inversely correlated with plasminogen activator inhibitor-1 antigen levels in hyperthyroid patients. *Endocrine Journal*.

[32] He G, Pedersen SB, Bruun JM, Lihn AS, Jensen PF, Richelsen B (2003). Differences in plasminogen activator inhibitor 1 in subcutaneous versus omental adipose tissue in non-obese and obese subjects. *Hormone and Metabolic Research*.

[33] Refsum H, Ueland PM, Nygård O, Vollset SE (1998). Homocysteine and cardiovascular disease. *Annual Review of Medicine*.

[34] Harker LA, Ross R, Slichter andScott SJCR (1976). Homocystine induced arteriosclerosis. The role of endothelial cell injury and platelet response in its genesis. *The Journal of Clinical Investigation*.

[35] Thampi P, Stewart BW, Joseph L, Melnyk SB, Hennings LJ, Nagarajan S (2008). Dietary homocysteine promotes atherosclerosis in apoE-deficient mice by inducing scavenger receptors expression. *Atherosclerosis*.

[36] Morris MS, Bostom AG, Jacques PF, Selhub J, Rosenberg IH (2001). Hyperhomocysteinemia and hypercholesterolemia associated with hypothyroidism in the third US National Health and Nutrition Examination Survey. *Atherosclerosis*.

[37] Nedrebø BG, Ericsson UB, Nygård O (1998). Plasma total homocysteine levels in hyperthyroid and hypothyroid patients. *Metabolism*.

[38] Diekman MJM, Van Der Put NM, Blom HJ, Tijssen JGP, Wiersinga WM (2001). Determinants of changes in plasma homocysteine in hyperthyroidism and hypothyroidism. *Clinical Endocrinology*.

[39] Nedrebø BG, Nygård O, Ueland PM, Lien EA (2001). Plasma total homocysteine in hyper- and hypothyroid patients before and during 12 months of treatment. *Clinical Chemistry*.

[40] Lindeman RD, Romero LJ, Schade DS, Wayne S, Baumgartner RN, Garry PJ (2003). Impact of subclinical hypothyroidism on serum total homocysteine concentrations, the prevalence of coronary heart disease (CHD), and CHD risk factors in the New Mexico Elder Health Survey. *Thyroid*.

[41] Durga J, Verhoef P, Bots ML, Schouten E (2004). Homocysteine and carotid intima-media thickness: a critical appraisal of the evidence. *Atherosclerosis*.

[42] Durga J, Bots ML, Schouten EG, Kok FJ, Verhoef P (2005). Low concentrations of folate, not hyperhomocysteinemia, are associated with carotid intima-media thickness. *Atherosclerosis*.

[43] Tsai MY, Arnett DK, Eckfeldt JH, Williams RR, Ellison RC (2000). Plasma homocysteine and its association with carotid intimal-medial wall thickness and prevalent coronary heart disease: NHLBI family heart study. *Atherosclerosis*.

[44] Danesh J, Collins R, Appleby P, Peto R (1998). Association of fibrinogen, C-reactive protein, albumin, or leukocyte count with coronary heart disease: meta-analyses of prospective studies. *JAMA*.

[45] Ridker PM, Hennekens CH, Buring JE, Rifai N (2000). C-reactive protein and other markers of inflammation in the prediction of cardiovascular disease in women. *The New England Journal of Medicine*.

[46] Blake GJ, Rifai N, Buring JE, Ridker PM (2003). Blood pressure, C-reactive protein, and risk of future cardiovascular events. *Circulation*.

[47] Folsom AR, Pankow JS, Tracy RP (2001). Association of C-reactive protein with markers of prevalent atherosclerotic disease. *American Journal of Cardiology*.

[48] Lowe GDO, Pepys MB (2006). C-reactive protein and cardiovascular disease: weighing the evidence. *Current Atherosclerosis Reports*.

[49] Pepys MB (2005). CRP or not CRP? That is the question. *Arteriosclerosis, Thrombosis, and Vascular Biology*.

[50] Sander D, Schulze-Horn C, Bickel H, Gnahn H, Bartels E, Conrad B (2006). Combined effects of hemoglobin A1c and C-reactive protein on the progression of subclinical carotid atherosclerosis: the INVADE Study. *Stroke*.

[51] Hashimoto H, Kitagawa K, Hougaku H, Etani H, Hori M (2006). C-reactive protein predicts carotid atherosclerosis progression in mild to moderate risk and middle-aged patients. *Clinical and Investigative Medicine*.

[52] Juonala M, Viikari JSA, Rönnemaa T, Taittonen L, Marniemi J, Raitakari OT (2006). Childhood C-reactive protein in predicting CRP and carotid intima-media thickness in adulthood: the Cardiovascular Risk in Young Finns Study. *Arteriosclerosis, Thrombosis, and Vascular Biology*.

[53] Lorenz MW, Karbstein P, Markus HS, Sitzer M (2007). High-sensitivity C-reactive protein is not associated with carotid intima-media progression: the Carotid Atherosclerosis Progression Study. *Stroke*.

[54] Toruner F, Altinova AE, Karakoc A (2008). Risk factors for cardiovascular disease in patients with subclinical hypothyroidism. *Advances in Therapy*.

[55] Nagasaki T, Inaba M, Shirakawa K (2007). Increased levels of C-reactive protein in hypothyroid patients and its correlation with arterial stiffness in the common carotid artery. *Biomedicine and Pharmacotherapy*.

[56] Pearce EN, Bogazzi F, Martino E (2003). The prevalence of elevated serum C-reactive protein levels in inflammatory and noninflammatory thyroid disease. *Thyroid*.

[57] Napoli R, Guardasole V, Zarra E (2010). Impaired endothelial- and nonendothelial-mediated vasodilation in patients with acute or chronic hypothyroidism. *Clinical Endocrinology*.

[58] Lee WY, Suh JY, Rhee EJ, Park JS, Sung KC, Kim SW (2004). Plasma CRP, apolipoprotein A-1, apolipoprotein B and Lp(a) levels according to thyroid function status. *Archives of Medical Research*.

[59] Christ-Crain M, Meier C, Guglielmetti M (2003). Elevated C-reactive protein and homocysteine values: cardiovascular risk factors in hypothyroidism? A cross-sectional and a double-blind, placebo-controlled trial. *Atherosclerosis*.

[60] Chu CH, Lee JK, Wang MC (2008). Change of visfatin, C-reactive protein concentrations, and insulin sensitivity in patients with hyperthyroidism. *Metabolism*.

[61] Burggraaf J, Lalezari S, Emei JJ (2001). Endothelial function in patients with hyperthyroidism before and after treatment with propranolol and thiamazol. *Thyroid*.

